# Regulation of the viability of *Nf1* deficient cells by PKC isoforms

**DOI:** 10.18632/oncotarget.2531

**Published:** 2014-09-26

**Authors:** Xiaodong Zhou, Ling Shen, Toshima Parris, Junchi Huang, Bo Yi, Khalil Helou, Changyan Chen

**Affiliations:** ^1^ Center for Drug Discovery, Northeastern University, Boston, USA; ^2^ The First Affiliated Hospital of Nanchang University, Nanchang, China; ^3^ The Institute of Clinical Sciences, Gothenburg University, Gothenburg, SE; ^4^ The Jiangxi Province Tumor Hospital, Nanchang, China

**Keywords:** PKC isoforms, NF1, ras, caspase 3, apoptosis

## Abstract

Suppression of protein kinase C (PKC) is known to be synthetically lethal with *ras* mutations in various types of cancer cells. The studies also showed that blockade of PKC affected the viability of *Nf1* deficient cells. Since PKC family consists of more than 10 isoforms, our study aimed at identifying which isoform(s) played the crucial role in sensitizing *Nf1* deficient cells to apoptosis. Using genetic and chemical PKC inhibitors, we demonstrated that the concurrent inhibition of PKC α and β induced *Nf1* deficient ST or 96.2 cells, but not SNF02.2 cells with a normal *Nf1* or ST cells ectopically expressing *Nf1* effective domain gene, to apoptosis. In this process, PKC δ in *Nf1* deficient cells, but not in ST/*Nf1* cells, was upregulated and translocated to the nucleus. Furthermore, caspase 3 was cleaved and cytochrome c was released to the cytosol. Thus, it appeared that PKC δ and α/β are the crucial components for sustaining the aberrant Ras signaling and further viability of *Nf1* deficient cells. The abrogation of these two isoforms activated their opponent PKC δ for switching on the caspase 3-governed apoptotic machinery.

## INTRODUCTION

Neurofibromin (the protein product of *Nf1*) shares sequence similarities with the catalytic domain of p120 Gap [1, 2]. Biochemical analysis of recombinant peptides corresponding to this domain and crystallography reveal that Nf1 is a GAP (GTPase-activating proteins) family member and negative regulator of Ras activation [1, 2]. Inactivation of Nf1 caused an increase in Ras activity in murine or some human tumor cells [3-5]. Thus, it appears that loss of function of Nf1 provided the growth advantage conferred by aberrant Ras signaling [1, 6-8]. The GAP activity of Nf1 has profound implications in the pathology and complications of Neurofibromatosis type 1. This common familial tumor predisposition syndrome is inherited in an autosomal dominant manner, and the common abnormality of this genetic defect is the formation of peripheral nerve tumors [9-12]. Approximate 30% of Neurofibromatosis type 1 patients develop malignant peripheral nerve sheath tumor (MPNST) that are often clinically resistant to conventional therapies.

PKC family consists of more than 10 isoforms that are serine/threonine protein kinases. These kinases differ in their structures, cellular functions and tissue distributions [13, 14]. PKC α, β_I_, β_II_ and γ are categorized as the conventional or classic PKC isoforms that are calcium- and diacylglycerol (DAG)-dependent for the activation, while isozymes of unconventional PKC subgroup (PKC δ, ε, η and θ) are independent of calcium for their functions. The atypical PKC isozymes (PKC ζ and λ/ν) require neither DAG nor calcium for being activated. The structural diversity and different tissue distributions render distinct specificities of PKC isozymes. Using small hairpin RNA (*shRNA*) and chemical inhibitors to disrupt individual PKC isoform or block the functions of each isoform *in vitro* and *in vivo*, it was shown that PKC isoforms are either pro- or anti-apoptotic, depending upon cell types, stimuli or contexts within signaling pathways [15-18.

Ras family consists of a group of small GTPases that regulate proliferation, differentiation, motility, transformation and apoptosis, via governing various downstream effectors. The active, GTP-bound form of Ras is able to interact with its downstream effectors and stimulate their activities. The balance among these intracellular signaling pathways is a key element to determine the fate of cells. It was demonstrated that, after the suppression of PKC, cells expression an aberrant *ras* were sensitized to apoptosis [19-21]. The effort for determining how oncogenic Ras transmitted pro-apoptotic signaling has been made [22-24].

Apoptosis is a major process for eliminating cancerous, damaged or un-wanted cells. Dysregulation of apoptosis has been directly linked to various diseases, including tumorigenesis. Studies indicate that the upregulation of apoptosis or re-sensitizing cells to apoptotic stimulation provides a promising potential for cancer therapies [25-27]. In many forms of apoptosis, caspase family members play the significant role in the initiation of cell death program. Caspases belong to a growing family of cysteine proteases and, all of them are synthesized as inactive proenzymes and being activated by proteolytic cleavage [28-30]. Among these isozymes, caspase 3 appears crucial in executing cell death program. During caspase cascade, the control of the mitochondrial transmembrane potential is disrupted, which permits the formation of permeability transition pores [28-30]. As a result of the increase in the permeability of the mitochondria membrane, cytochrome c is released to the cytosol and apoptosis is induced.

In this study, we demonstrate that mutated *Nf1*, together with the concurrent loss of PKC α and β, are synthetically lethal. In this lethal process, PKC δ was activated, accompanied with capase 3 cleavage and the releasing of the mitochondrial cytochrome c to the cytosol. Thus, our data suggested that PKC α and β play critical roles in maintaining the homeostasis as well as viability of *Nf1* deficient cells.

## RESULTS

### HMG treatment sensitizes *Nf1* deficient cells to apoptosis

**Figure 1:**
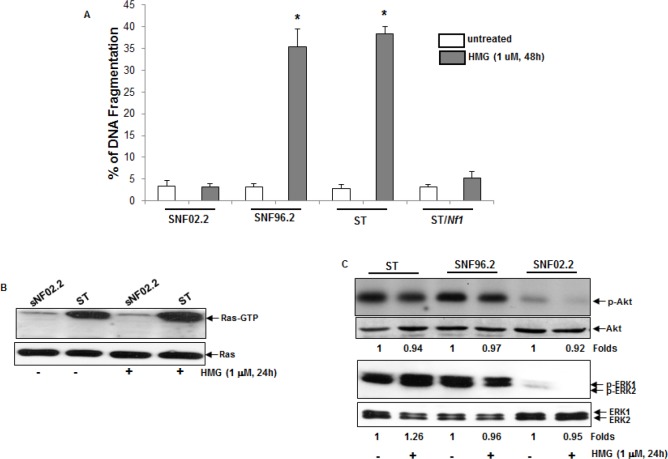
Inhibition of PKC triggered apoptosis in *Nf1* deficient cells A. SNF02.2, SNF96.2, ST and ST/*Nf1* cells were treated with HMG (1.0 μM) for 48 h, and then subjected to DNA fragmentation assay. The error bars are SD (standard deviation) over 5 independent experiments (n = 5, *, *p* < 0.005). B. Cell lysates were isolated from the cells for Ras Pull-Down assay. The even loadings of the cell lysates were normalized by ras expression. C. With or without HMG treatment, cell lysates were isolated and then immunoblotted with the anti-phosphor-Akt or –ERK1/2 antibody. The even loadings of the cell lysates were normalized by Akt or ERK1/2 expression. The changes of protein expression levels in untreated- vs. treated-cells were measured, which were presented as n-folds.

After the suppression of endogenous PKC, cancer cells harboring oncogenic *ras* became susceptible to programmed cell death [19-21]. *Nf1* deficient cells have been suggested to be sensitive to PKC inhibitor [20, 31]. Therefore, human *Nf1* deficient ST or SNF96.2 cells and SNF02.2 cells expressing a functional *Nf1* or ST/*Nf1* cells (that were stably transfected with the *Nf1* effective domain gene) were treated with HMG (1-O-methyl-rac-glycerol, a PKC inhibitor) and the occurrence of apoptosis was analyzed by DNA fragmentation assay (Figure 1A). Forty-eight hours after the treatment, approximate 40% of ST or sNF96.2 cells underwent apoptosis. In comparison, only a few of treated SNF02.2 or ST/*Nf1* cells became apoptotic. Annexin V-FITC apoptotic detection assay was also conducted. A similar result was obtained (data not shown). It indicated that PKC inhibition was able to sensitize *Nf1* deficient cells to apoptosis.

Next, the active status of Ras or its downstream effectors in *Nf1* deficient cells was analyzed. After the addition of HMG, the amount of GTP-bound Ras was tested by the active Ras pull-down and detection kit. A high level of Ras was bound to GTP in ST cells and HMG treatment did not further increase Ras activation (Figure 1B). A baseline expression of GTP-bound Ras was detected in untreated or HMG-treated SNF02.2 cells. Akt and ERK1/2 often function downstream of Ras and have been implicated in regulating the growth promotion under *Nf1* deficient conditions [4, 32]. Thus, the phosphorylation status of these Ras downstream effectors was analyzed by immunoblotting. A high amount of the phosphorylation form of Akt or ERK1/2 was detected in ST or SNF96.2 cells, which were not further increased by HMG treatment. Consistently, the activation of these Ras effectors was undetectable in SNF02.2 cells with or without HMG treatment (Figure 1C). The phosphorylation status of JNK or p38 in these cells was also tested. It appeared that JNK or p38 was not active in *Nf1* deficient cells with or without HMG treatment (data not shown). The results indicated that Ras and some of its downstream effectors were active under *Nf1* deficient condition, but the inhibition of PKC by HMG did not further affect Ras regulated signaling.

### Concurrent knockdown of PKC α and β was responsible for the induction of apoptosis in *Nf1* deficient cells

**Figure 2:**
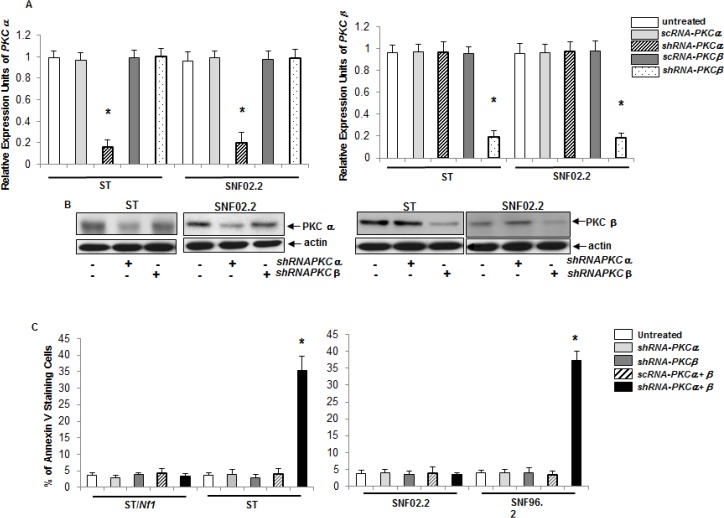
Induction of apoptosis by the concurrent knockdown of *PKC α* and *β* A. ST or SNF02.2 cells were transiently infected *sc* or *shRNA-PKC α*, *β* for 48 h, and *mRNAs* from the cells were prepared for real time-PCR analysis. The error bars are SD over 5 independent experiments (n = 5, *, *p* < 0.005). B. The cells were infected with *shRNA-PKC α* or *β*, lysed and immunoblotted with the antibodies for the expression of PKC α or β. Actin was used as loading control. C. After the transient infection of *sc* or *shRNA-PKC α*, *β* and both for 48 h, the cells were subjected to Annexin V-FITC analysis. The error bars represent SD over five independent experiments (n = 5, *, *p* < 0.006).

To further test the role of PKC in the initiation of this apoptotic process, we constructed the *shRNA*s targeting the conventional subgroup of PKC isoforms as well as corresponding scrambled *shRNAs*, using a lentiviral vector. After *sc* or *shRNAPKC α* or *β* were transiently infected into ST or SNF02.2 cells, the levels of *α* (left panel) and *β* (right panel) expression were measured by real-time PCR analysis (Figure 2A). The infection of *shRNAPKC α* or *β*, but not the *scRNAs*, effectively knocked down the expressions of the corresponding genes. The protein expressions of these two PKC isoforms, after the transient infection of *shRNAPKC α* or *β*, were also examined by immunoblotting (Figure 2B). The amount of PKC α or β was dramatically reduced after the infection of the *shRNAs*, respectively. The knockdown effects of other conventional PKC isoforms (such as PKC ε, θ) were also tested. Consistently, the *shRNAs*, but not *scRNAs* were able to efficiently interfere with the gene or protein expressions of the corresponding *PKC* isoforms (data not shown).

The concurrent knockdown of PKC α and β was shown to induce apoptosis in various type of cells ecotopically expressing *v-ras* or cancer cells harboring oncogenic *ras* [19-21]. Therefore, the induction of apoptosis in *Nf1* proficient or deficient cells, after the knockdown of *PKC α*, *β* or both, was tested using Annexin V-FITC apoptotic detection assay (Figure 2C). The infection of *shRNAPKC α*, *β* or *scRNAPKC α* + *β* had no effect on the viability of all cell lines. However, the concurrent suppression of *PKC α* and *β* triggered apoptosis in more than 35% of ST or SNF96.2 cells. It appeared in a good agreement with that mediated by HMG (see figure 1A). To confirm this, DNA fragmentation assay was also conducted. After the concurrent suppression of PKC α and β by the *shRNA*s, a similar result was obtained (data not shown). The induction of apoptosis in the cells after knockdown of other conventional PKC isoform alone or in pairwise combinations was also analyzed. None of these isoforms alone or in various paired-combinations had a profound effect on *Nf1* deficient cells for the induction of apoptosis (data not shown).

### PKC δ was activated in the absence of PKC α and β under *Nf1* defect condition

**Figure 3:**
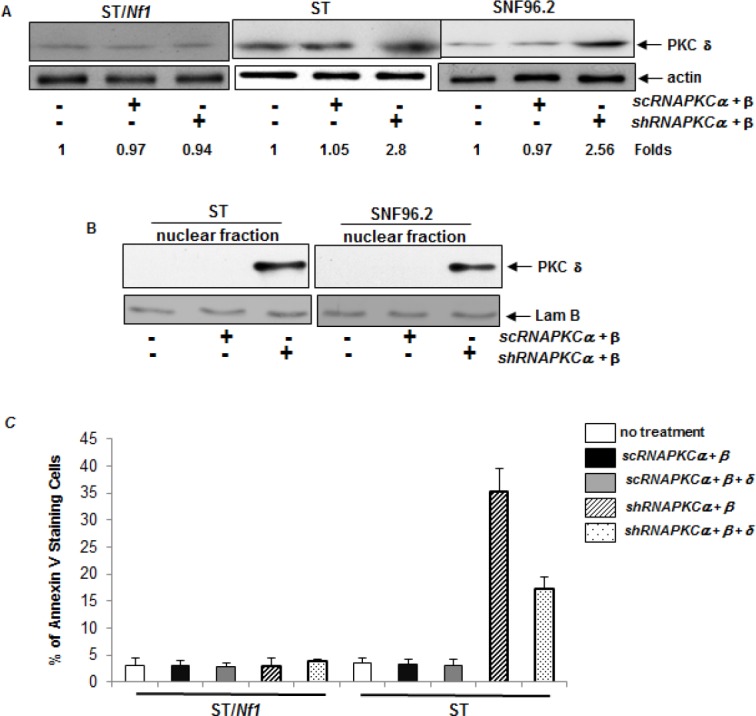
PKC δ participated in the regulation of the apoptotic process A. *Nf1* deficient ST or SNF96.2 cells were co-infected with either *sc* or *shRNA-PKC α* and *β*, and then immunoblotted for PKC δ expression. The relative expression level of proteins was normalized by actin expression. The changes of protein expression levels in the cells with or without being infected with the *shRNAs* were measured, which were presented as n-folds. B. After the co-knockdown of *PKC α* and *β*, the nuclear fraction was isolated from the cells and immunoblotted with anti-PKC δ antibody. Lamin B was used for the loading controls. C. After the transient co-infection of *sc* or *shRNA-PKC α*/*β* or triple-infection with *sc-* or *shRNA-PKC δ*, the cells were subjected to Annexin V-FITC analysis. The error bars represent SD over five independent experiments (n = 5, *, *p* < 0.005).

PKC δ was shown to be either pro- or anti-apoptotic, depending upon the circumstances, cell types or nature of stimuli [16, 17]. It is also known that PKC δ perturbed the growth promotion mediated by PKC α and β in cancer cells harboring aberrant *ras* [20]. Thus, the expression of PKC δ in *Nf1* deficient or proficient cells was examined after the co-infection of *sc* or *shRNA-PKC α* and *β* (Figure 3A). The level of PKC δ was unchanged in all cells after the infection of the *scRNAs*. In comparison, the level of this kinase was significantly increased in ST or SNF96.2 cells after the co-knockdown of *PKC α* and *β*.

The active PKC δ is able to translocate to the nucleus, because it possesses a NLS (nuclear localization sequence) domain, and further initiates cell death program there.^33^ This led us to test the subcellular localization of PKC δ in our experimental setting (Figure 3B). After co-infected with *shRNA-PKCα* and *β*, the nuclear fractions of ST and SNF96.2 cells were isolated. PKC δ was detected by anti-PKC δ antibody in the *Nf1* deficient cells after the co-knockdown of *PKC α* and *β*. However, this kinase was absent in the nuclear fraction of either untreated cells or the cells infected with the *scRNAs*. The data indicated that PKC δ was activated and translocated to the nucleus in the absence of both PKC α and β in *Nf1* deficient cells.

Subsequently, the role of PKC δ in the induction of apoptosis in *Nf1* deficient cells was tested (Figure 3C). ST/*Nf1* and ST cells were co-infected with *sc*, *shRNA-PKC α/β*, or triple-infected with the *sc*, *shRNA-PKC δ*. The occurrence of apoptosis of the cells was then analyzed by Annexin V-FITC assay. Again, approximate 40% of ST cells, but not ST/*Nf1* cells, became apoptotic. After the knockdown of PKC δ, the magnitude of apoptosis in the absence of PKC α and β in ST cells was partially blocked. The data suggested that PKC δ was one of the potential players in the induction of apoptosis in *Nf1* deficient cells when PKC α and β are concurrently knocked down.

### Caspase 3 was activated and participated in this lethal reaction

**Figure 4:**
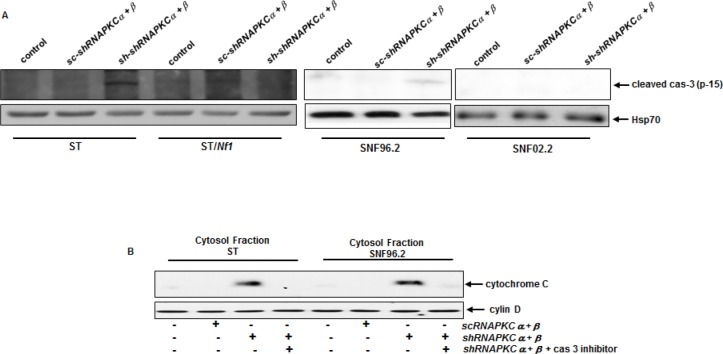
Activation of caspase 3 in *Nf1* deficient cells after the co-knockdown of PKC α and β A. After co-infection of *sc* or *shRNA-PKC α* and *β*, cell lysates were isolated and immunoblotted for the expression of active caspase 3. Heat shot protein 70 was used for the loading control. B. After the co-knockdown of PKC α and β, the cytosolic fraction from ST or SNF96.2 cells were isolated in the presence or absence of caspase 3 inhibitor, and then immunoblotted with anti-cytochrome c antibody. Cyclin D1 is a G_1_/S cell cycle regulator and located in the cytosol. Thus, this protein was used for the loading control.

Caspase family members are the major factors for the execution of cell death program and caspase 3 often serves as a common executor in apoptosis [28-30]. To determine if caspase 3 was activated in our experimental setting, the cleavage of caspase 3 was analyzed by immunoblotting. The active, cleaved form of caspase 3 was detected in ST or SNF96.2 cells after the co-knockdown of *PKC α* and *β*, which was absent in SNF02.2 or ST/*NF1* cells following the same treatment (Figure 4A). The cleaved form of caspase 3 was absent in SNF02.2 or ST/*NF1* cells with or without HMG treatment. Since caspase activation often leads to the release of cytochrome c from the mitochondria to the cytosol [34, 35], the existence of cytochrome c in the cytosol of ST and SNF02.2 cells with or without the co-knockdown of *PKC α* and *β* was analyzed by immunoblotting (Figure 4b). Indeed, cytochrome c was present in the cytosol of the *Nf1* deficent cells in the absence of PKC α and β, which was blocked by the addition of caspase 3 inhibitor. The co-infection of *shRNA-PKC α* and *β* did not induce the cytosol release of cytochrome c in *Nf1* proficient cells. The data suggested that caspase 3 was crucial for cytochrome c releasing in this apoptotic process.

**Figure 5:**
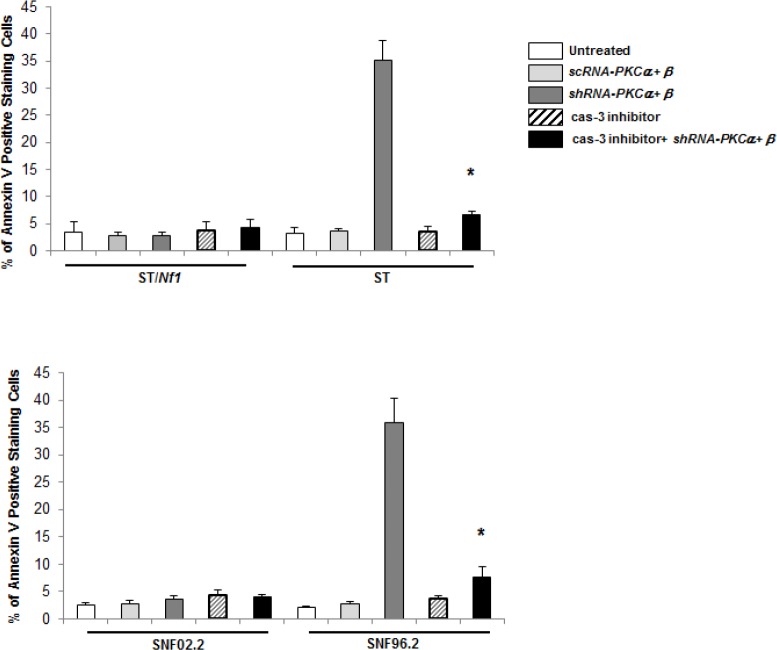
Inhibition of caspase 3 blocked the induction of apoptosis elicited by *PKC α* and *β* knockdown in the *Nf1* deficient cells Following the co-infection of *sc* or *shRNA-PKC α* and *β*, in the presence of absence of caspase 3 inhibitor, the *Nf1* deficient cells were subjected to Annexin V-FITC analysis. The error bars represent SD over five independent experiments (n = 5, *, *p* < 0.005).

To further confirm the role caspase 3 in the induction of apoptosis observed here, Annexin V apoptotic detection assay was in the presence or absence of caspase 3 inhibitor (Figure 5). As expected, the co-knockdown of PKC α and β sensitized ST or SNF96.2 cells, but not sNF02.2 or ST/*Nf1* cells, to apoptosis. The addition of caspase 3 inhibitor blocked this apoptotic process in *Nf1* deficient cells.

## DISCUSSION

The study of Ras mitogenic signaling has been the popular theme in the field of cancer research in light of its pivotal role in the regulation of diverse cellular activities, including proliferation, differentiation, senescence and apoptosis. In tumorigenesis, hyperactive Ras signaling is supported or modulated by its downstream effectors or parallel factors for maintaining the balance of the deregulated signaling and accordingly increased metabolic rates of cancer cells. Disruption of the supporting factors or pathways would perturb the homeostasis in cancer cells harboring mutated ras and further trigger an apoptotic crisis. Recently, targeting pivotal supporting factors of oncogenic Ras has been viewed as an important strategy for development of new anti-cancer therapies. Studies including ours demonstrated that mutated *ras*, together with loss of PKC, were synthetically lethal [19-21]. Although it is known that *Nf1* deficient cells were sensitive to PKC inhibitors, little is known about the underlying mechanisms. In this study, using genetic PKC inhibitors, we identified that PKC α and β both were critical for the regulation of the viability of *Nf1* deficient cells. We showed that the concurrent knockdown of PKC α and β triggered apoptosis in the cells with *Nf1* defect, in which PKC δ acted as a tumor suppressor for inducing apoptosis. In this process, caspase 3 was activated and cytochrome c was released from the mitochondria to the cytosol. Our study suggested that PKC α and β were crucial for supporting hyperactive Ras signaling under *Nf1* deficient condition and potential targets for therapeutic intervention of neurofibromatosis type 1, especially MPNST.

Ras is hyper-activated under the condition of *Nf1* deficiency. In order to maintain the deregulated Ras signals or to keep high metabolic rates driven by aberrant Ras, a coordination of the Ras signaling with other parallel pathways may be pivotal. Disruption of one or more of these signaling pathways would perturb the homeostasis of *Nf1* deficient cells and further trigger an apoptotic catastrophe. Studies showed that in the absence of PKC, cancer or transformed cells expressing oncogenic *ras* were unstable and rapidly underwent apoptosis [19-21]. Here, our study revealed that the concurrent knockdown of α and β was fatal to *Nf1* deficient cells. Since *Nf1* defects can be detected in the majority of MPNST patients, it would be tempting to target PKC α and β for clinical intervention of this devastating disease.

As serine/threonine protein kinases, PKC isoforms are structurally distinct and functionally diverse. The functions of PKC isoforms have been shown to be often dependent upon different cellular contexts or types of stimuli. For example, studies demonstrated that PKC isoforms could either positively or negatively regulate either tumorigenesis or apoptosis under certain circumstances, indicating the functional complexity of PKC isozymes [16, 36]. The interconnection between PKC and Ras signaling pathways was reported [37, 38]. Upon mitogenic stimulation, the SH2 binding sites of PKC are phosphorylated, in concomitant with the recruitment of Grb2/SOS and activation of Ras in T lymphocytes [38]. PKC, via modulating Ral activity, was also shown to negatively regulate Ras signaling [37]. Many studies suggested that PKC δ is a tumor suppressor and functioned opposite of other phorbol ester dependent PKC isoforms [20, 33, 39, 40]. Following the co-suppression of PKC α/β, PKC δ in *v-ras* transformed murine fibroblasts or cancer cells harboring oncogenic *ras* was activated and exerted its anti-tumor action via interacting with p73 [20]. It is likely that PKC (especially PKC α and β) copes with hyper-active Ras signaling to promote the growth of *Nf1* deficient cells. Once the growth preference supported by PKC α and β is perturbed, an apoptotic process is elicited, which may be due to unleash the negative control rendered by PKC δ. It is also conceivable that cross-talks between PKC isoforms and other intracellular signal transducers exist in a hierarchic or parallel order within different subcellular compartments. Once cross-talks are perturbed, a catastrophe erupts.

Using the inhibitor that predominantly suppresses the phorbol ester and calcium dependent isoforms of PKC (especially PKC α and β), we have demonstrated that a mitotic checkpoint was activated in a Chk1-dependent fashion, which further triggered mitotic catastrophe in *Nf1* deficient cells [41]. It is known that deregulated mitotic regulators often initiated a persistent mitotic arrest, resulting in mitotic slippage and cell death [42]. It is possible that the active PKC δ in our experimental setting phosphorylates Chk1 and further activates mitotic exit checkpoint, leading to an apoptotic crisis. The investigation to explore the underlying mechanisms by which PKC δ elicits the mitotic checkpoint to eliminate *Nf1* deficient cells after the co-knockdown of PKC α and β is under way.

In summary, our study showed that the concurrent knockdown of PKC α and β sensitized the cells with *Nf1* deficiency to undergo apoptosis, which appeared due to hyperactive Ras or its aberrant downstream effector signaling. PKC and Nf1 are important intracellular signal transducers and play crucial roles in the regulation of cell differentiation and proliferation. Deregulated PKC signaling and mutated Nf1 alone is compatible with cell viability. However, mutations of *Nf1*, together with loss of PKC (in particular PKC α plus β), severely perturb survival signaling pathways, resulting in eliciting an apoptotic crisis in the cells. With increasing attention in targeting aberrant Ras or its cooperative signaling for cancer treatment, our study provided the information for developing new therapeutic strategies that preferentially kill tumors with *Nf1* defect at clinically achievable doses.

## METHODS

### Cells and reagents

Human *Nf1* deficient ST, SNF96.2 or proficient SNF02.2 cells were purchased from ATCC (Manassas, VA). The cells were cultured in Dulbecco's Modified Eagles's medium supplemented with 10% heat-inactivated Fetal Bovine Serum (Atlanta Biologicals), 100 units/ml penicillin, 100μg/ml Streptomycin (Invitrogen). ST cells were stably transfected with the *Nf1* effective domain gene and maintained in the medium containing 400 μg/ml of G418 (Fisher Scientific Inc. MA). HMG was purchased from EMD Millipore (Billerica, MA). Antibodies were purchased from BD (San Jose, CA).

The oligonucleotides containing small interference RNA sequences targeting different Protein Kinase C isozymes were ligated to a lentiviral small-hairpin (sh) RNA expression vector pLentiLox3.7. The sequences of the *shRNA α* and *β* are: *5′-ggctgtacttcgtcatgga-3′* and *5′-caggaagtcatcaggaata-3′* for human PKC α and PKC β, respectively. FuGene 6 transfection reagent (Roche Applied Science, IN) was used for transfections.

### DNA fragmentation analysis

DNA fragmentation data was collected by a flow cytometer and analyzed by the Cell-Fit software program (BD Biosciences). Cell-Fit receives data from the flow cytometer and provides real-time statistical analysis, computed at one second intervals. Cells with sub-G_0_-G_1_ DNA contents after staining with propidium iodide were counted as apoptotic cells. In brief, following treatments, cells were harvested and fixed in 70% cold ethanol. Afterwards, cells were stained with 0.1 μg/ml propidium iodide containing 1.5μg/ml RNase. DNA contents of cells were then tested by a flowcytometer.

### Annexin V-FITC apoptosis detection assay

After treatments, cells were prepared and stained with Annexin V-FITC Apoptosis Detection Kit I (BD Biosciences, CA) according to manufacturer's instructions. Subsequently, the samples were analyzed by a flow cytometer.

### Ras activation assay

Active Ras Pull-Down and Detection kit (Thermo. Scientific, IL) was used. The analysis of GTP-bound Ras was performed according to the protocol provided by the company.

### Immunoblot analysis

Cell lysates were separated by SDS-PAGE gel and transferred to nitrocellulose. After blocking with 5% non-fat milk for 1 hour at room temperature, the nitrocellulose was probed with corresponding antibodies and then visualized by chemilluminescence (Perkin-Elmer, MA).

### Real-Time PCR

Total RNAs were isolated and reversely transcribed. cDNAs were used for real-time PCR analysis. *β-actin* was used as the control. The primers were as follows: *5′-agaagggcacatcaaaatcg-3′* and *5′-acgcccaccaatctacagac-3′* for *PKC α*; 5′-ctccattcctgcttccagac-3′ and *5′-aacagaccgatggcaatctc-3′* for *PKC β*.

### Caspase 3 activity assay

A caspase 3 assay kit (Biovision, CA) was used to analyze the activity of caspase 3 in the cells. Briefly, after the treatments, cell lasates were prepared and the activity of caspase 3 was then analyzed.

### Preparation of the subcellular fractions

The cytosol and nuclear fractions were isolated using the kit from BioVision (Milpitas, CA). Briefly, cells were incubated with 1% Triton X-114 lysis buffer (1% Triton X-114, 25 mM Tris, pH7.5, 20 mM MgCl_2_, 150 mM NaCl, 1 μg/mL aprotinin and 1 μg/mL leupeptin) on ice for 30 min and then homogenized by passing through a 25-gauge needle for 45 passages. After centrifuging at 280 *g* for 15 min, supernantant was collected as the cytosol fraction. The precipitated nuclei were then lysed with nuclear lysis buffer (50 mM Tris-Cl, pH7.6, 10 mM EDTA, 1% SDS, 1 mM PMSF, 1 μg/ml aprotinin and 1 μg/ml leupeptin) on ice for 10 min. The nuclear fraction was collected by re-centrifuging at 280 *g* for 15 min.

### Statistical analysis

Averages and standard deviations of the results of the experiments were computed. Standard deviations are displayed as error bars in the figures. A Student's T test was used and a *p* value of <0.005 was considered very significant.
